# Evolution of the unspliced transcriptome

**DOI:** 10.1186/s12862-015-0437-7

**Published:** 2015-08-20

**Authors:** Jan Engelhardt, Peter F. Stadler

**Affiliations:** Bioinformatics Group, Department of Computer Science, and Interdisciplinary Center for Bioinformatics, University of Leipzig, Haertelstraße 16-18, Leipzig, D-04107 Germany; Max Planck Institute for Mathematics in the Sciences, Inselstraße 22, Leipzig, D-04103 Germany; Fraunhofer Institut for Cell Therapy and Immunology, Perlickstraße 1, Leipzig, D-04103 Germany; Institute for Theoretical Chemistry, University of Vienna, Währingerstrasse 17, Vienna, A-1090 Austria; Center for non-coding RNA in Technology and Health, University of Copenhagen, Grønnegårdsvej 3, Frederiksberg, 1870 Denmark; Santa Fe Institute, 1399 Hyde Park Rd., Santa Fe, 87501 NM USA

## Abstract

**Background:**

Despite their abundance, unspliced EST data have received little attention as a source of information on non-coding RNAs. Very little is know, therefore, about the genomic distribution of unspliced non-coding transcripts and their relationship with the much better studied regularly spliced products. In particular, their evolution has remained virtually unstudied.

**Results:**

We systematically study the evidence on unspliced transcripts available in EST annotation tracks for human and mouse, comprising 104,980 and 66,109 unspliced EST clusters, respectively. Roughly one third of these are located totally inside introns of known genes (TINs) and another third overlaps exonic regions (PINs). Eleven percent are “intergenic”, far away from any annotated gene. Direct evidence for the independent transcription of many PINs and TINs is obtained from CAGE tag and chromatin data. We predict more than 2000 3’UTR-associated RNA candidates for each human and mouse. Fifteen to twenty percent of the unspliced EST cluster are conserved between human and mouse. With the exception of TINs, the sequences of unspliced EST clusters evolve significantly slower than genomic background. Furthermore, like spliced lincRNAs, they show highly tissue-specific expression patterns.

**Conclusions:**

Unspliced long non-coding RNAs are an important, rapidly evolving, component of mammalian transcriptomes. Their analysis is complicated by their preferential association with complex transcribed loci that usually also harbor a plethora of spliced transcripts. Unspliced EST data, although typically disregarded in transcriptome analysis, can be used to gain insights into this rarely investigated transcriptome component. The frequently postulated connection between lack of splicing and nuclear retention and the surprising overlap of chromatin-associated transcripts suggests that this class of transcripts might be involved in chromatin organization and possibly other mechanisms of epigenetic control.

**Electronic supplementary material:**

The online version of this article (doi:10.1186/s12862-015-0437-7) contains supplementary material, which is available to authorized users.

## Background

Non-coding RNA (ncRNA) constitutes a significant portion of the mammalian transcriptome [1–4]. Although a large sub-class of long ncRNAs (lncRNAs) is spliced, capped, and polyadenylated, and thus differs from their protein-coding siblings only in coding capacity [5], these mRNA-like lncRNAs account for only a small fraction of the striking diversity of transcripts. Nuclear retained ncRNAs are often spliced transcripts but not polyadenylated. These “*dark matter* RNAs”, which have remained largely un-annotated so far, can in fact be the dominating non-ribosomal RNA component in a mammalian cell [6, 7].

As a class, lncRNAs are under purifying selection [8–10] although the level of sequence conservation is typically very low [9, 11]. Comparative transcriptomics [12, 13] as well as computational studies [14, 15] showed that between one and two thirds of human lncRNAs are conserved among Eutheria, emphasizing the functional importance of these transcripts. The catalogs of RNAs used in these studies are, however, heavily biased towards spliced RNAs, and some of them use the conservation of splice sites explicitly as a means to assess conservation [12, 15–17]. Indeed, the overwhelming majority of lncRNAs for which detailed functional information is available is spliced, see e.g. [18], although splicing often tends to occur only after transcription and is less efficient [19].

By far the largest class of unspliced transcripts for which a function is known are the intron-less protein-coding genes. In human, they account for about 4.5 % of the protein-coding loci [20]. They have received comparably little attention even though they have distinctive features e.g. related to their export pathways [21]. On average they are expressed at lower levels, tend to be more tissue specific, evolve at faster rates, and are of relatively recent origins [22]. The extremely well-conserved replication-dependent histone genes form a distinctive class of intron-less genes set apart by their unique 3’ end processing [23].

Unspliced lncRNAs fall into at least four distinct classes: (i) intronic transcripts typically associated with protein-coding loci, (ii) lncRNAs associated with long 3’-UTRs, (iii) independent unspliced RNAs found in intergenic regions, and (iv) an enigmatic class of very long macroRNAs.

Totally and partially intronic transcripts (TINs and PINs) that are usually unspliced and lack coding capacity have been reported in large numbers for both human and mouse [24–26]. This class includes many unspliced long anti-sense intronic RNAs [27, 28]. Not much is known on the biogenesis of intronic lncRNAs, although there are presumably multiple pathways. The anti-sense TIN ANRASSF1 is a pol-II transcript, capped, and polyadenylated [29]. Sense TINs, on the other hand, might also be processing products generated from introns as described for the sisRNAs in frog oocytes [30]. Intronic transcripts appear to have primarily regulatory functions, for which they employ several different molecular mechanisms [29, 31, 32]. LncRNAs can affect gene expression both *in cis* and *in trans* by modulating the chromatin structure [33]. Although the first reports that RNA is an integral component of chromatin date back to the 1970s [34] there has not been much work on chromatin-associated RNAs (CARs). They are largely non-polyadenylated [35] and derive from both intronic and intergenic regions [36]. Some of them are probably natural antisense transcripts (NATs) deriving from both coding and non-coding loci that act *in cis* as epigenetic regulators of gene expression and chromatin remodeling [37]. Furthermore RNP complexes stably tethered to chromatin were characterized recently that appear to play a role in regulating higher order structures of chromatin [38]. Many promoter-associated long ncRNAs [39] might also function via chromatin modification [40].

The 3’ untranslated regions (3’UTRs) of eukaryotic genes are well known to harbor sequence and secondary structure elements that regulate mRNA stability, localization, and translation [41–43]. Many 3’UTRs in animals can also be decoupled from the protein-coding part forming so-called UTR-associated RNAs (uaRNAs), which appear to function as non-coding RNAs in trans [44]. Note that the abbreviation uaRNA was later also used for the unrelated class of “upstream antisense RNAs” in [45]. In the form of independent uaRNAs, they are often detectable as unspliced ESTs. A recent study, furthermore, found parallels in sequence composition between lncRNAs and 3’ UTRs that sets both groups apart from 5’UTRs and coding regions [46]. Since 3’UTRs typically harbor microRNA target sites they may function as sponges for microRNAs [47, 48] or RNA binding proteins [49].

The best-known examples of “intergenic” unspliced ncRNAs are MALAT-1 and MEN *β*, which organize nuclear structures known as SC35 speckles and paraspeckles, respectively [50–52]. Both transcripts are spliced only infrequently [53] and are rather well-conserved [54]. This class also contains important disease-associated RNAs such as PRNCR1 [55].

MacroRNAs covering up to several hundred kb were recently observed as highly expressed RNAs in signaling pathways [56] and in cancer cells [6]. Very similar transcripts such as Airn [57, 58] and KCNQ1OT1 [59] are involved in the regulation of imprinted loci. These enigmatic RNAs are very poorly conserved.

Despite the wealth of unspliced EST data that is available in public databases, they have received little attention as a source of information on non-coding RNAs apart from a seminal work [24]. In part this is probably due the difficulties involved in mining this resource. First, strand information is usually not available and the absence of splice junctions makes it impossible to guess the reading direction. Furthermore, a detailed analysis of nearly 40,000 putative ncRNAs from RIKEN’s FANTOM transcript data set showed that many of the putative intronic and intergenic transcripts might be artifacts and in fact are internally primed subsequences from even longer transcripts [60]. The fragmentary nature of ESTs, furthermore, makes it difficult and often impossible to reliably determine transcript boundaries.

In this contribution we use unspliced EST data to obtain an overview of the unspliced transcripts in the human and the mouse genome. We focus on ESTs rather than NGS data here primarily because the much longer EST sequences are largely mapped unambiguously to the genome and are much more likely to contain splice junctions when they originate from spliced transcripts than the much shorted NGS reads. We update and extend previous investigations in the human uEST data [24, 61] and in particular provide a first overview of the similarities and differences of the situation in human and mouse.

## Results and discussion

### ESTs “within range” of RefSeq genes

The majority of unspliced EST (uEST) clusters, 89 % in both human and mouse, overlaps with or lies in the vicinity of annotated RefSeq genes. A possible reason for this strong association with known pre-existing annotation could be that uEST cluster are just by-products of “normal” spliced transcripts arising from occasionally inefficient splicing or a background of not yet processed primary transcripts. We therefore compare the amount of spliced and unspliced ESTs with the range of annotated RefSeq genes (including a 5 kb flanking region), see Additional file 1: Figure S1 for human and Additional file 1: Figure S2 for mouse. The amount of ESTs is a measure that interpolates between diversity of tissues and conditions under which the locus is expressed (for low counts) and a genuine proxy for expression levels (for large counts). Despite its aggregate nature, the correlation of the amount of spliced and unspliced ESTs is indicative of the overall coupling between spliced and unspliced expression. We observe a Pearson correlation coefficient of *r*=0.75 and *r*=0.5 for spliced and unspliced RefSeq genes in human and *r*=0.72 and *r*=0.48 in mouse gene, respectively. The correlation coefficients are rather moderate, however, reaching roughly the level of correlation between mRNA and protein abundances, see e.g. [62, 63]. We take this as a strong indication that the observed unspliced EST clusters are not just a byproduct. The quantitative data suggest that a considerable fraction of unspliced transcripts is independent of spliced genes.

**Fig. 1:**
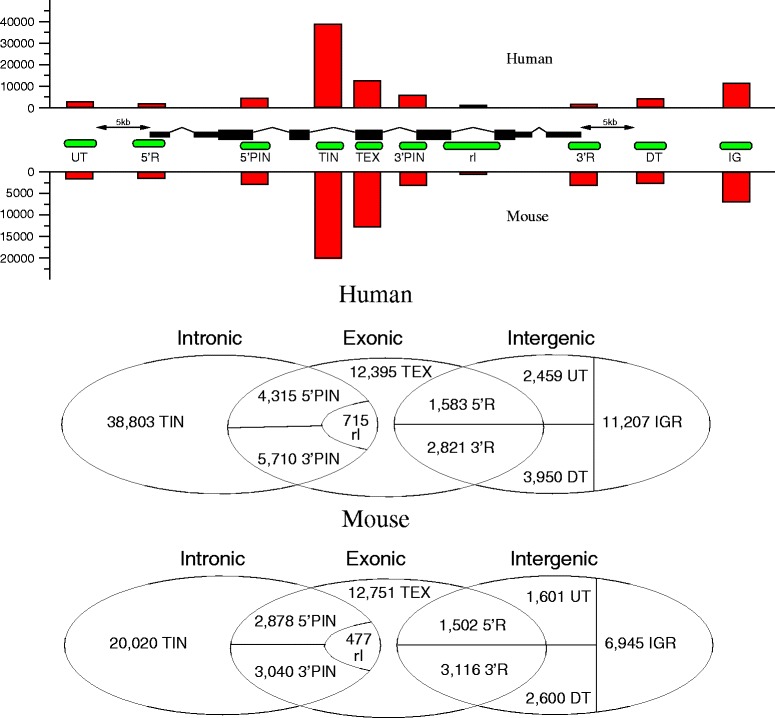
Classification of unspliced EST cluster w.r.t. their location relative to RefSeq genes. With the exception of totally intronic RNAs (TINs) and cluster in the upstream (UT) and downstream (DT) region within 5 kb, all other classes partially overlap RefSeq exons: 5’ and 3’ partially intronic RNAs (5’PIN, 3’PIN), EST cluster overlapping 3’UTR and downstream region (3’R) or 5’UTR and upstream region (5’R), resp., and cluster covering complete introns indicating retained introns (rI) are distinguished in the statistical analysis. Furthermore, we record totally exonic cluster (TEX) and the intergenic clusters (IGR) that are unrelated to RefSeq loci. The bar plots above and below the scheme summarize the numbers of unspliced EST for each cluster type in human (above) and mouse (below). The Venn diagram below lists the exact numbers. About one fifth of the unspliced EST clusters (21,022 in human and 11,179 in mouse) cannot be classified unambiguously because they are overlapped by more than one RefSeq gene and would fall into different classes with respect to these, see subsection Classification in the Methods part for details. These ambiguous clusters are not included here

We analyzed the relative location of unspliced ESTs and components of RefSeq genes in detail to elucidate their relationships, see Fig. 1. Compared to a similar analysis ([24], Table 1) we now a have much larger data set of *Totally INtronic* (TIN) unspliced EST cluster, comprising 38,803 (vs. 5678) but nearly the same amount of *Partially INtronic* (PIN), 10,015 (vs. 9132). The number of reported TINs has increased by a factor of 6.8, while the known PINs increased only by about 10 %, suggesting that the coverage of TINs is much farther from saturation than that of the PINs. While [24] and our previous study [61] were concerned with the human transcriptome only, we consider here a comparable data set for the mouse (*Mus musculus*).

**Table 1:** Human and mouse unspliced EST clusters overlapping a predicted TSS peak in their putative 5’ region and their orientation relative to the surrounding RefSeq gene. RefSeq TSS are all unspliced EST cluster that overlap any 5’UTR or that are located completely within the upstream region of a RefSeq gene. TINs and PINs are defined as in Fig. 1. All unspliced EST cluster that have an overlap with the 3’UTR but not with the 5’ UTR are interpreted as uaRNAs. “Sense” and “Antisense” are relative to the reading direction of the RefSeq gene. “Ambiguous” clusters could not be assigned to an orientation because of conflicting information on their directions. The column “All” lists the number of corresponding unspliced EST clusters without considering an overlap with CAGE-tags

Species	Human				Mouse			
Type	Sense	Antisense	Ambiguous	All	Sense	Antisense	Ambiguous	All
RefSeq TSS	3829	696	1269	12,337	4010	533	1307	9556
TINs^a^	1924	703	212	38,803	1082	255	132	20,020
5’ PINs^a^	880	15	116	2926	256	16	46	1527
3’ PINs^a^	312	119	73	5439	171	64	46	2902
uaRNAs	1833	246	709	17,331	1501	201	592	17,126

^a^unspliced EST cluster overlapping 5’ UTR have been excluded for this analysis

The protein-coding part of RefSeq genes overlaps 39,791 unspliced EST cluster (27,897 in mouse). Less than 4 % of these cluster in each species, however, are located completely in the coding region. We analyze not only uESTs overlapping the gene body but take into account the vicinity of RefSeq genes, defined as within 5 kb of the ends of the RefSeq entry. There are 2459 (1601) unspliced EST cluster in upstream region of human (mouse) RefSeq genes and 3950 (2600) downstream, see Fig. 1. Conversely, 37,561 of 42,165 (89 %) of the annotated RefSeq genes (ignoring separately annotated haplotypes) overlap with at least one unspliced EST cluster.

### Independent UTR-derived RNAs

Unspliced EST (uEST) cluster are predominately expressed in the 3’ region of RefSeq genes. There are more than 6000 human and 5000 mouse uEST clusters that overlap the 3’UTR or are located within 5000 nt downstream of 3’end of a RefSeq genes. The frequent observation of these downstream signals is consistent with the observation that the length of UTRs is often underestimated in current gene annotations [64]. Alternative polyadenylation, and hence often dramatic changes in 3’UTR length, furthermore appear to be frequent phenomenon that may have biased the current gene annotations [65–67]. Unfortunately, we can not rule out that unspliced EST clusters of the class 3’R are only artifacts of the read-through of transcripts from the upstream gene. Therefore, we attempt to use CAGE data to gain additional evidence of the 3’R cluster to be independently transcribed or processed, for instance similar to uaRNAs.

CAGE tags [68] are routinely used to assay CAPed 5’ ends. The FANTOM 5 consortium predicted TSS peaks by clustering CAGE tags [69]. These peaks can be used to predict the reading direction of an unspliced EST cluster if they are located at one of the ends of the cluster. 30 % (human) and 25 % (mouse) of the predicted TSS peaks (319,019 of 1,048,124 in human and 169,144 of 652,833 in mouse) overlap with a total of 48,435 (human) and 30,056 (mouse) uEST clusters.We are primarily interested in the TSS peaks from the forward strand that are located close to the beginning of the EST cluster, and in the TSS peaks from the reverse strand that are located close to the end of the EST cluster. The reading direction of an EST cluster can be determined unambiguously provided it contains exactly one of these two types of TSS peaks. As in previous work [61], we use 84 nt from either end of the EST cluster as distance cutoff. We obtained 9378 forward and 8837 reverse strand uEST cluster in human and 6732 forward and 6630 reverse in mouse. For 918 cluster in human and 719 mouse we found TSS peaks at both ends. These cases are likely bi-directionally transcribed loci. We nevertheless excluded them from further analysis, summarized in Additional file 1: Table S3.

Most of them correspond to the 5’ end or to 5’ extensions of RefSeq genes. We excluded them in the further analysis since we can not distinguish the transcription start site (TSS) of the corresponding RefSeq gene and an independent one of an unspliced EST cluster, see Table 1.

The majority (6957/9877, 70 % in human; 3841/5593, 69 % in mouse) of the remaining clusters, share the reading direction of the corresponding gene. Some of these unspliced cluster may correspond to a class of transcribed promoter-associated ncRNAs similar to the CCND1-pncRNAs [40], which negatively regulate CCND1 transcription by recruiting TLS to the promoter.

Enhancer RNAs are a class of non-coding RNAs that has recently gained considerable interest [70–73]. They are expressed from enhancer regions. Since these are not annotated as (RefSeq) genes, we analyzed whether intergenic unspliced EST cluster might correspond to enhancer RNAs. To this end we used a set of 38,554 predicted human enhancer RNAs compiled in [74] as part of the FANTOM 5 project. However, only 281 of 11,207 human IGR uEST cluster overlap with one of these enhancer RNAs.

### Cell-specific expression

The CAGE data collected by the FANTOM consortium can even be used to analyze the cell-specific expression of unspliced EST (uEST) cluster. *Ohmiya et al*. [75] used the FANTOM 5 dataset to predict transcriptions start sites (TSSs) in 156 different human primary cells. Overlapping these TSS with the uEST clusters provides us with an overview of cell-specific expression patterns of many uEST cluster.

For 6766 uEST cluster on the forward and 6106 on the reverse strand a cell-type-specific expression can be detected. Most clusters (58 %) are expressed in less than 10 cell lines, see Table 2 and Additional file 1: Figure S5. In total there are 8221 uEST cluster overlapping or being in close vicinity to a gene and 501 from intergenic regions (4150 cluster could not be assigned to a class, see Fig. 1 and description). For the more cell-type specific cases (equal or less than 10 cell lines) their relative distribution is quite similar. On the other end of the spectrum there are 575 (7 %) gene-associated uEST cluster expressed in equal or more than 147 cell lines. There is only a single intergenic cluster which is expressed in 147 cell lines. This is not surprising since very abundant transcripts are unlikely to have escaped all efforts to annotate human genes.

**Table 2:** Amount of unspliced EST clusters that are expressed in a certain number (1st column) of cell lines. The number in brackets is the relative amount in respect to all unspliced EST clusters of that group for which a predicted TSS was present. Percentages below 5 % are omitted. “Genic” uEST cluster are overlapping or in close vicinity of RefSeq genes

#Cell lines	“Genic” uEST	IGR uEST	All uEST
10	113	8	175
9	144	7	215
8	137	8	220
7	183	12	274
6	198	14	296
5	240	25(5 %)	387
4	326	27 (5 %)	499
3	479 (6 %)	26 (5 %)	726 (6 %)
2	897 (11 %)	65 (13 %)	1337 (10 %)
1	2236 (27 %)	158 (32 %)	3319 (26 %)

We performed the same analysis for all RefSeq genes as well as long non-coding RNAs (lncRNAs) from GENCODE v14 filtered for high reliability, see Additional file 1. The distribution of RefSeq genes is similar to the unspliced EST cluster, although more uEST clusters are expressed in very few tissues and there are fewer nearly ubiquitously expressed uEST clusters compared to protein coding genes. The lncRNAs lack the peak for ubiquitous expression completely, consistent with previous observations that lncRNAs are expressed less abundantly and more specifically compared to traditional transcripts [76].

We tried to use the cell-specific CAGE data to find cell types with an enrichment for uaRNAs. Using all 156 cell types, normalized for the amount of predicted TSS per cell type, there is, however, no enrichment detectable for any particular cell type. We then combined related cell types into larger groups but, again, no group-wise enrichment’s were visible.

#### Chromatin architecture

CAGE is a method developed for exactly determining the 5’-end of RNAs. A more indirect way to detect the transcription start site (TSS) of a gene is analyzing the chromatin structure.

We used a genome-segmentation track based on ENCODE data provided by ref. [77] based on a computational methods for unsupervised chromatin state annotation from ChIP-seq data for multiple histone modifications, general transcription factors, and chromatin accessibility assays. While the chromatin elements were computed independently for six different cell lines in [77] we combined all cell lines here since the definition of the EST clusters is also a composite of many different cell and tissue types.

More than 80 % of the clusters close to or within annotated genes but outside of exons (i.e., TIN, UT, DT) overlap a segment predicted to be transcriptionally active (‘TSS’ or ‘Transcribed’) based on chromatin state in at least one cell line. Although we know, of course, that unspliced EST clusters are transcribed regions, it is reassuring that the chromatin data show a consistent picture. In particular for UT and DT uEST clusters it is impossible to discern in most cases whether they are independent transcripts or processing products, or whether they belong to large, splice transcripts whose UTRs are annotated only incompletely.

Small inaccuracies in gene annotation are not relevant for unspliced EST cluster in intergenic region (IGR) which are at least 5000nt away from the next annotated gene. In total 7714 intergenic cluster overlap regions annotated to be transcriptionally active. Seven hundred and thirty-three with Words of them intersect predicted ‘TSS’, see Table 3 for detailed count statistics.

**Table 3:** Unspliced EST (uEST) cluster overlapping with chromatin elements predicted from ENCODE data [77]. ‘TSS’ represents “Predicted promoter region including TSS”. ‘Transcribed’ refers to “Predicted transcribed region”. ‘All’ is the number of all uEST cluster of this class with the relative amount of cluster that have an overlap with ‘TSS’ or ‘Transcribed’ in parentheses

TYPE	TSS	Transcribed	All (%)
TIN	3406	34,709	38,803 (92.6 %)
UT	1068	1278	2459 (82.8 %)
DT	203	3390	3950 (87.3 %)
3R	227	2601	2821 (93.3 %)
IGR	733	7299	11,207 (68.8 %)

#### UTR-associated RNAs

UTR-associated RNAs (uaRNA) are identified by accumulations of CAGE tags, i.e., transcription start sites (TSS) or re-capping sites, that are independent of the underlying genes. Detectable uaRNAs overlap with the 3’UTR of their surrounding gene. Conceivably, 5’-uaRNAs might also exist. However, it is nearly impossible to distinguish the TSS (or alternative TSSs) of the surrounding gene from the 5’-end of an embedded uaRNA. In contrast, CAGE tags towards the 3’-end of a transcript exclude most or all of the coding sequence or the major part of a lncRNA, hence strongly suggesting a distinct transcript. Operationally, uaRNA candidates are defined as unspliced EST clusters that overlap a predicted TSS and the 3’UTR of a RefSeq gene but not its 5’UTR. Using TSS predicted by chromatin data as described above we identify in total of 1547 bona fide uaRNA candidates in human. Relying on TSS predicted by the FANTOM 5 consortium using CAGE tags [69] there are 2788 uaRNA candidates in human and 2294 in mouse. Four hundred and twelve of the uaRNA candidates in human are detected simultaneously by chromatin data and CAGE data. Of these, 160 have an ortholog unspliced EST cluster in mouse. Unfortunately, comprehensive chromatin data sets are not available for mouse.

### Unannotated intergenic EST clusters

11,207 human and 6945 mouse uEST clusters are located more then 5 kb away from any RefSeq gene. In human 4986 of these overlap non-RefSeq annotated mRNAs. Removing these leaves 6222 human and 3412 mouse candidates of novel, predominantly unspliced genes.

Recent retropseudogenes [78] may lead to mapping artifacts since ESTs might be mapped erroneously to the intron-free locus instead of a spliced alignment to the locus of the functional gene. Although revived retrogenes exist and can be functional [79] they cannot be distinguished reliably from mapping artifacts. Hence we compared the sequences of the cluster against the “Retroposed Genes, Including Pseudogenes”-track from UCSC genome browser in order to identify and remove such cases from further analysis. Seven hundred and sixty loci (12 %) of the candidates are identified as deriving from protein-coding genes. In the mouse data set, the relative number is higher: 864 (25 %) of the candidates show significant similarities from coding genes. The majority of the remaining unknown transcripts are probably long extensions of 3’UTRs of spliced genes. In many of these cases we might be seeing uaRNAs.

To further investigate the 5462 intergenic unspliced EST cluster that were not recognized as likely retrogenes, we used RNAz 2.1 [80, 81] to detect signatures of purifying selection on RNA secondary structure and RNAcode [82] to find evidence for conserved protein-coding regions. We used multiple genome alignments taken from the UCSC genome browser as input. This data set covers 4,059,185 nt and yields 1104 RNAz hits with a classification probability *P*_*RNAz*_>0.5 and 371 with *P*_*RNAz*_>0.9. In mouse the 1,550,680 nt are covering 249 RNAz hits with a classification probability *P*_*RNAz*_>0.5 and 77 with *P*_*RNAz*_>0.9. Compared to the predicted 6880 low confidence and 2259 high confidence hits in the 30 Mb long ENCODE regions [81], the human result amounts to a very moderate enrichment by a factor of 1.2. It remains unclear whether this enrichment is confounded e.g. by the restriction of unspliced EST cluster to genomic regions with relatively high expression. The enrichment of structured RNA elements could be interpreted as hinting at a function of long unspliced regions in specific binding with proteins. An example for a conserved secondary structure element is shown in Additional file 1: Figure S7.

Using RNAcode, we detected 632 regions with a p-value *p*_*RNAcode*_<0.01 in human. Two hundred and thirty-two of these regions have a length of at least 60 nucleotides. In mouse there are 652 regions with a p-value *p*_*RNAcode*_<0.01. Two hundred and sixty-five have a length of at least 60 nt. Shorter regions mostly appear to be artifacts caused by short segments of coding region in retrogenes.

Overlapping all unspliced EST cluster with a recent version of GENCODE lncRNAs (v22 and vM5) we noticed that 15.1 % of human and 8.6 % of mouse uEST cluster overlap with annotated lncRNAs. Interestingly, human intergenic cluster are enriched in this set (3281, i.e., 29 % of all IGR uEST cluster). In mouse, the 642 IGR cluster (9 %) overlapping GENCODE lncRNAs closely matches the relative amount of IGR uEST cluster. The specific increase in human might be due to a better annotation compared to mouse.

Human intergenic unspliced EST cluster are the class with the most cluster that are not found to be expressed by the ENCODE RNA-seq data [83]. In total, 96.6 % of all human unspliced EST cluster are supported by RNA-seq data, defined as being covered by at least 10 reads of ENCODE RNA-seq data. 1723, of 3519 cluster in total that are not covered, belong to the class IGR, the numbers for all classes can be found in Additional file 1: Table S4. This indicates that this class contains a higher amount of low expressed genes that are more difficult to detect.

### Conservation

#### Pairwise orthology

**Fig. 2:**
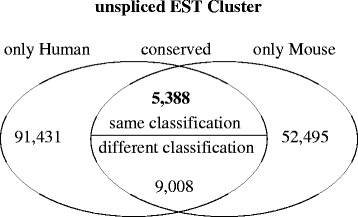
Using the pairwise alignments of human and mouse we could detect 14,396 pairs of unspliced EST cluster which are conserved. The pairs consist of 13,278 different cluster from human and 13,277 from mouse. Five thousand, three hundred and eighty-eight pairs are between cluster which are classified in the same class, see Fig. 1 for details about classification. 91,431 (87 %) of 104,980 unspliced EST cluster in human and 52,495 (80 %) of 66,109 in mouse can not be associated with an homologous unspliced EST cluster in the other species

The first publication on unspliced EST analysis [61] focused just on the human genome. The motivation for including mouse data is to get an evolutionary perspective. After detecting unspliced EST cluster in both, human and mouse, we are able to predict evolutionary conserved ones. Unfortunately, for most parts of the data we could not find a homologous cluster. This is either the case if there is no homologous region detected by liftOver, see subsection Conservation in the Methods part for more information, or if there is no unspliced EST cluster in the homologous region. Both cases sum up to 87 % of human and 80 % of the mouse unspliced EST cluster, see Fig. 2. There are 14,396 unspliced EST cluster conserved between human and mouse. Of the conserved cluster, 5388 are classified in the same class, according to Fig. 1, 9008 belong to a different class in both species, see Additional file 1: Figure S8.

Unsurprisingly, the class Totally EXonic (TEX) comprises the most conserved cluster (2464). One reason for that is the fact that detection of conservation between already known genes is much easier. The same goes for Totally INtronic (TIN) cluster, see Additional file 1: Figure S9 for an example. However, we also found 73 conserved intergenic cluster. One example can be seen in Additional file 1: Figure S10. 42 of the conserved uEST cluster in human and 28 in mouse overlap regions previously shown to have enhancer activity from the *VISTA Enhancer Browser*. The conservation between human and mouse implicitly hints at functional transcripts that have to be protected from harmful mutations.

#### Sequence conservation

A more global view on the sequence conservation of the unspliced EST cluster can be gained by using multiz alignments from the UCSC genome browser. In our analysis we use both the 100way-alignment with human as reference and the 60way mouse-centered alignment. For both of them phastCons scores [84, 85], which indicate the level of conservation of the reference species, are available through the UCSC genome browser. The phastCons scores, which are in the range between 0 and 1, represent probabilities of negative selection. The underlying model corrects for base composition.

**Fig. 3:**
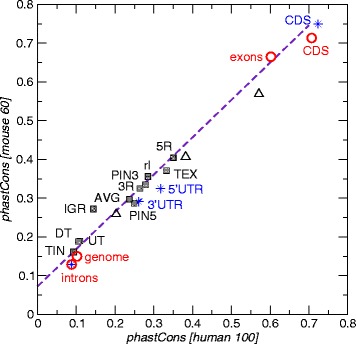
Mean phastCons scores for the different classes of uEST clusters (squares), compared to the average conservation of introns, the entire genome and (predominantly coding) exons. AVG is the average of all uEST classes. TEX uESTs are particularly heterogeneous, hence they were also subdivided into three subclasses indicated as triangles (completely in CDS, partially overlapping CDS, and entirely non-coding from upper right to lower left). Blue stars refer to a background set comprising only those RefSeq genes in which we detected uEST clusters

To be able to interpret the results for unspliced EST cluster we also analyzed the average sequence conservation of introns, the entire genome and exons, as well as a background set comprising all those RefSeq genes in which we detected uEST clusters. Most classes of unspliced EST cluster exhibit an increased level of average sequence conservation compared to the genomic background, see Fig. 3. In the case of TEX cluster one should notice that only a minority of them (human: 1446/12,395; mouse: 820/12,751) is located completely in coding sequence (CDS). The remaining part is just partly or not at all overlapping CDS. This explains the lower average phastCons score of TEX compared to exons. A small increase in conservation levels is visible in particular also for 5R and 3R uESTs compared to the annotated 5’- and 3’-UTRs in the RefSeq-based background set. Only for TINs we could not detect a significant extra conservation. However this might be due to the difficulties of defining a comparable genomic background considering the fact that the conservation of introns is quite heterogeneous. Figure 3 also conveys a comparison of the situation in human and mouse, showing the sequence conservation levels are globally very similar between human and mouse.

## Conclusions

Unspliced EST data, although typically disregarded in transcriptome analysis, can provide interesting insights into the structure of human transcriptomes. They outline a major component of transcriptional output that totally or at least partially escapes splicing. Nevertheless, this valuable resource has not been mined comprehensively in the past. So far, only the partially and totally intronic transcripts (PINs and TINs), which constitute more than half of the clusters of unspliced ESTs, have received attention in systematic studies [24–26]. Our analysis confirmed the conclusions drawn from the literature on these abundant class of transcripts in human and mouse. In addition, we find direct evidence of the independent transcription of PINs and TINs based on CAGE tag and chromatin data. Several promising candidates for previously unknown long RNAs could be found.

Many of the unspliced EST clusters form extensions of the UTRs of well-annotated genes. On the 5’-side they provide additional information about the transcriptional start sites (TSS) of the annotated RefSeq genes themselves. Not surprisingly, the majority of clusters with clear support for a TSS from CAGE data fall into this class. More interestingly, however, the relative majority of UTR extensions is located at the 3’-end of RefSeq genes and forms typically extensive, several kb-long extensions. In line with [44], many of these 3’UTR extensions contain a TSS for an independently transcribed uaRNA. We identify 2788 likely uaRNAs in human and 2294 in mouse with strong support from the CAGE data. In addition 1547 uaRNA candidates in human were detected using chromatin data. Four hundred and twelve candidates in human were predicted by both methods and 160 of them have an ortholog cluster in mouse.

CAGE data was also used to analyze the expression in 156 different primary human cell types. Most of the unspliced EST cluster are expressed in less than 10 different cell types, while only a small fraction of very abundant ones in expressed in all cell types. This distribution is similar to that of known RefSeq genes but more skewed towards specifically expressed loci.

This suggest that hundreds or even thousands of the 3’UTR extensions are EST clusters overlapping the 3’UTR of a RefSeq gene but originate rather from independent transcripts than a single 3’UTR. In fact, 227 cluster overlapping 3’UTR and downstream region of a gene and a similar number of DT uEST clusters carry histone marks characteristic for TSS, see Table 3. In addition, it is demonstrated in [86] that developmental-stage- and tissue-specific cleavage and subsequent secondary capping of 3’UTR is not an infrequent phenomenon. The production of such unspliced RNAs may explain the 3R uEST clusters that have CAGE tags but no promoter-like histone features.

Although TINs and PINs have been reported to be transcribed mostly independently of their surrounding gene, only a small fraction has a sufficient concentration of CAGE tags for a recognizable TSS. However there are more than 3000 human TINs overlapping a chromatin mark indicating a TSS.

The remaining set of unspliced EST clusters outside a 5 kb range around RefSeq genes comprises 11 % of the data. About a tenth (a forth in mouse) of these clusters overlaps retrogenes and retropseudogenes. For these cases, it is often impossible to distinguish between truly expressed loci and mapping artifacts of ESTs arising from the spliced original of the gene. The manual inspection of a random sample of the remaining cases shows that a large fraction of these EST clusters constitute even larger extensions of 3’UTRs. In many cases they also appear to be long 3’UTRs or uaRNAs belonging to previously unannotated, typically non-coding, spliced transcripts.

To date little is known about the evolutionary age of long ncRNAs. In our study only 15–20 % of the unspliced EST cluster are conserved between human and mouse. This fraction is lower than the numbers reported for spliced lncRNAs [12–15]. It nevertheless indicates a substantial level of conservation that is corroborated by elevated levels of sequence conservation in most subclasses of uEST clusters. Ignoring technical limitations preventing us from detecting a larger number, this pattern of conservation suggests a species or lineage-specific function of the remaining transcripts. The 5388 pairs of conserved unspliced EST clusters with the same classification in human and mouse are likely to be functionally important and we expect them be present also in other mammalian species.

In summary, the analysis of unspliced ESTs uncovers a largely unexplored realm of long transcripts. Including many previously unknown transcripts. The frequently postulated connection between lack of splicing and nuclear retention, see e.g. [87] suggests that this class of transcripts might be involved in chromatin organization and possibly other mechanisms of epigenetic control. Further experimental investigation of the long transcripts could provide great insight into these mechanisms.

## Methods

### Data

The annotation track ‘all_est’ for human genome assembly hg19 was downloaded from the UCSC genome browser (30th of April 2013) [88]. Starting from ‘ESTs including unspliced’ we removed all ESTs with more than 30 deleted nucleotides compared to the reference genome. This gets rid of all the annotated spliced ESTs contained in this track. It also discards sequences with sometimes long, intron-like, gaps that are not annotated as spliced ESTs because they map without canonical splice sites. The cutoff of 30 nucleotides was chosen since even smaller introns are extremely rare [89, 90]. We furthermore discarded ESTs mapped with more than 5 % mismatches. The hg19 release of the human genome contains 9 alternative haplotypes for highly variable regions on chromosome 6 [91]. They were not included in the further analysis to prevent counting identical ESTs several times. We obtained 3,425,788 unspliced ESTs (Table 4). The same procedure was used for the ‘all_est’ track for mouse genome assembly mm10, again downloaded from the UCSC genome browser (30th of April 2013). We obtained 2,177,648 unspliced ESTs in mouse (Table 4).

**Table 4:** Summary of human EST data. Analysis of the EST annotation track of hg19 and mm10 downloaded from the UCSC genome browser in April 2013. The numbers indicate successive filters. Which means just the ESTs which do not have introns larger than 30 nucleotides are included in the number of introns with more than 5 *%* mismatches and so on

Type	Human	Mouse
All ESTs	8,675,182	4,370,322
ESTs on Haplotypes	534,970	n/a
> 30*n* *t* gaps	4,514,773	2,107,660
> 5 *%* mismatches	169,967	68,239
Overlap with NUMTs	29,684	16,775
Unspliced ESTs	3,425,788	2,177,648
Unspliced EST cluster	104,980	66,109

A possible source of contamination is nuclear encoded mitochondrial DNA (NUMT) [92]. Since the mitochondrial transcripts remain unspliced at least in mammals [93], it is impossible to reliably distinguish unspliced ESTs mapping to recent NUMTs from fragments of mitochondrial transcripts. An annotation track for human and mouse NUMTs was recently provided in [94].

The gene locations and structure were taken from the ‘RefSeq Genes’ track [95] downloaded from the UCSC genome browser. Multiple genome alignments in maf format for human (46way) and mouse (60way) have also been downloaded using the UCSC table browser [88]. Input alignments for RNAcode have to be filtered for alignments with a length of less than 21 nucleotides for computational reasons. These alignments were discarded. The genome segmentation based on ENCODE data for all six cell lines (GM12878, H1-hESC, K562, HeLa-S3, HepG2, HUVEC) was downloaded from the ENCODE public hub at the UCSC genome browser (8th of January 2014). The combined segmentation was used. Coordinates of chromatin-associated RNAs (CARs) were taken from the supplemental material of [36] and lifted over to hg19.

Long non-coding RNA data was downloaded from the GENCODE web page [96] for the version v22 in human and vM5 in mouse. Enhancer RNAs were downloaded from the supplement of *Anderssonet al.*[74] in the FANTOM web browser [97]. The robust set of transcribed enhancers was used.

### Software

For most of the analyses which checked if a set of unspliced EST cluster overlaps another set of genomic intervals *bedtools* [98] (v2.20.1) were used. If the data was present the functionality of the Table Browser integrated in the UCSC genome browser was used. For the classification a custom perl script was developed. All statistical analyses were performed using the standard R function (R version 3.0.2) [99]. The Vienna RNA package was used in version 2.1.9, RNAz in version 2.1 and RNAcode in version 0.3. Programs were combined using standard bash and awk commands. Additional information on used commands and scripts is available on request.

### Analysis pipeline

We define unspliced ESTs that overlap each other or that are separated by at most 30nt as members of the same cluster. In order to avoid artifacts of both the experimental and the computation procedures we only consider unspliced EST cluster comprising at least three individual ESTs, leaving 104,980 human and 66,109 mouse unspliced EST cluster for further analysis. ESTs overlapping a NUMT have been discarded before the cluster analysis. In order to computer overlaps with existing annotation we used the functionality of the Table Browser integrated in the UCSC genome browser and the “Operate on Genomic Intervals”-tools of the Galaxy Browser [100] as well as BEDTools [98].

### Classification

In order to gain insights about the location of unspliced EST (uEST) cluster relative to RefSeq gene components we classified the uEST cluster in different classes. To a lesser extent this was also done by *Nakayaet al.*, 2007 [24] who introduced the terms TIN (totally intronic) and TEX (totally exonic). For every uEST cluster the class for every overlapping RefSeq gene and a 5 kb upstream/downstream region was determined to be one of the following classes:

- TEX - Overlap exclusively with a RefSeq exons including UnTranslated Regions (UTR) and non-coding RNA
- TIN - Overlap exclusively with a RefSeq intron
- 5’PIN - Overlap exclusively with an adjoining exon-intron pair, where the exon is located upstream of the intron
- 3’PIN - Overlap exclusively with an adjoining exon-intron pair, where the exon is located downstream of the intron
- rI - Overlap exclusively with one intron and both adjacent exons
- 5’R - Overlap with a RefSeq exon and the 5 kb upstream region but not with downstream region or 3’UTR
- 3’R - Overlap with a RefSeq exon and the 5 kb downstream region but not with upstream region or 5’UTR
- UT - Overlap exclusively with 5 kb upstream region
- DT - Overlap exclusively with 5 kb downstream region
- IGR - No overlap with any RefSeq gene or the 5 kb upstream/downstream region
- NO_CLASS - None of the other classes could be applied

If the uEST cluster was determined to have the same class for all overlapping RefSeq genes, it was assigned to this class. In case of conflicting data it was assigned to the class “NO_CLASS”. An exception was made if the conflict was between one of the classes TEX, TIN, 5’ PIN, 3’ PIN, rI, 5’R and 3’R and one of the classes UT or DT. In this case the overlap with an additional upstream/downstream region was ignored. Otherwise a class assignment would be impossible in regions with a high gene density. To avoid misclassifications due to small mistakes in the borders of RefSeq gene components the overlap between the uEST cluster and the gene component had to be more than 20 nucleotides to be considered. Note that the classes are not overlapping each other. Every unspliced EST cluster is assigned to exactly one class, with all ambiguous cases collected in “NO_CLASS” an excluded from all class-specific analyses.

#### CAGE analysis

TSS peaks predicted by FANTOM 5 for the hg19 and mm9 assemblies were downloaded from the FANTOM 5 public hub in the UCSC genome browser. The mm9 coordinates were converted to mm10 using the UCSC liftOver tool [101]. We used the permissive set. If an unspliced EST cluster overlaps with an predicted forward TSS peak in its first 84 nucleotides but not with an reverse TSS peak it is defined as a forward oriented transcript. If a cluster overlaps a TSS peak on the opposite strand in its last 84 nucleotides but not with a forward TSS peak its defined as reverse oriented transcript. Unspliced EST cluster which are detected as forward and reverse oriented simultaneously are disregarded.

The cell-specific CAGE data generated by FANTOM 5 was downloaded from the ’Fantom Web Resource’ [97, 102]. These comprise the supplementary data of ref. [75]. The same analysis as above was performed individually for all 156 cell types.

#### Chromatin architecture analysis

Annotation for six different cell lines (GM12878, H1-hESC, K562, HeLa-S3, HepG2, HUVEC) are available. We took all cell lines together for this analysis. Every position in the genome was assigned to one of the following classes: Predicted promoter region including TSS (TSS), Predicted promoter flanking region (PF), Predicted enhancer (E), Predicted weak enhancer or open chromatin cis regulatory element (WE), CTCF enriched element (CTCF), Predicted transcribed region (T) and Predicted Repressed or Low Activity region (R).

#### ENCODE RNA-seq data

The full set of ENCODE RNA-seq data [83] was downloaded from the EBI web server (http://ftp.ebi.ac.uk/pub/databases/ensembl/encode/integration_data_jan2011/byDataType/rna_signal/jan2011/hub/). It contains 351 bigWig-files which were converted to the format bedGraph using bigWigToBedGraph, a utility from the UCSC genome browser website. BedGraph can be used as an input for bedtools. All individual files were intersected with the list of unspliced EST cluster using *bedtools intersect*. The output consisted of one file for each of the 351 RNA-seq libraries containing the amount of reads overlapping each unspliced EST cluster. We reported the numbers of unspliced EST cluster that overlapped with at least 10 reads in at least one library.

### Pairwise orthology

For detecting conserved regions we use already existing pairwise alignments between human and mouse generated by the UCSC/Penn State Bioinformatics comparative genomics alignment pipeline [103]. The human-mouse alignment was created by aligning both genomes using blastz [104]. The coordinates were afterwards corrected using in-house scripts. The mouse-human alignment was done by using chainSwap “to translate hg19-reference blastz alignment to mm10 into mm10-referenced chains aligned to hg19.” [101]. Interestingly the mm10-based alignment leads to more ortholog clusters than the original one.

The chain-files were used as input to liftOver [101], a tool original developed to switch between genome assemblies. The human unspliced EST cluster were lifted over to mm10 by using the hg19-reference alignment, mouse unspliced EST cluster were lifted over to hg19 by using the mm10-referenced alignment, respectively. We combined all ortholog pairs detected by one of the liftOver runs.

### Sequence conservation

The multiz 100way-alignment with human as reference and the 60way mouse-centered alignment from UCSC Genome Browser was used for this analysis. For every unspliced EST cluster the average phastCons score [84, 85] for the region was determined using the UCSC table browser [88]. The phastCons scores represent probabilities of negative selection in a range from 0 to 1. They are derived from the two-state phylo-HMM and are defined as the posterior probability that the corresponding alignment column was generated by the conserved state (rather than the non-conserved state) of the phylo-HMM, given the model parameters and the multiple alignment [84]. The phastCons score also considers the flanking columns in an alignment. The average score per cluster was used to visualize the mean phastCons score per class in Fig. 3.

In case of cluster in intergenic regions, the ones with a significant RNAcode signal have been removed. The full phastCons statistic can be found in Additional file 1: Table S5.

## Additional file

Additional file 1
**Supplement.** Supplemental information about the methods and results. Includes example regions showing unspliced ESTs cluster detected to be especially interesting for various reasons. **Additional data – UCSC Track Hubs**; A track hub for the UCSC genome browser was created. It consists of a track of all unspliced EST cluster as well as tracks for the different classes of unspliced EST cluster for hg19 and mm10. Additional bed files for intermediate results of the analysis are available on request. The track can be accessed at: http://www.bioinf.uni-leipzig.de/~jane/data/uESTHub/hub.txt. Additional information how to use it can be found in the UCSC manual pages: http://www.genome.ucsc.edu/goldenPath/help/hgTrackHubHelp.html. (PDF 1587 kb)

## References

[1] The ENCODE Project Consortium (2007). Identification and analysis of functional elements in 1 % of the human genome by the ENCODE pilot project. Nature.

[2] Maeda N, Kasukawa T, Oyama R, Gough J, Frith M, Engström PG (2006). Transcript annotation in FANTOM3: Mouse Gene Catalog based on physical cDNAs. PLoS Genet.

[3] Clark MB, Amaral PP, Schlesinger FJ, Dinger ME, Taft RJ, Rinn JL (2011). The reality of pervasive transcription. PLoS Biol.

[4] The ENCODE Project Consortium (2012). An integrated encyclopedia of DNA elements in the human genome. Nature.

[5] Guttman M, Garber M, Levin JZ, Donaghey J, Robinson J, Adiconis X (2010). Ab initio reconstruction of cell type-specific transcriptomes in mouse reveals the conserved multi-exonic structure of lincRNAs. Nat Biotechnol.

[6] Kapranov P, St Laurent G, Raz T, Ozsolak F, Reynolds CP, Sorensen PH (2010). The majority of total nuclear-encoded non-ribosomal RNA in a human cell is ‘dark matter’ un-annotated RNA. BMC Biol.

[7] St Laurent G, Vyatkin Y, Kapranov P (2014). Dark matter RNA illuminates the puzzle of genome-wide association studies. BMC Med.

[8] Ponjavic J, Ponting CP, Lunter G (2007). Functionality or transcriptional noise? Evidence for selection within long noncoding RNAs. Genome Res.

[9] Marques AC, Ponting CP (2009). Catalogues of mammalian long noncoding RNAs: modest conservation and incompleteness. Genome Biol.

[10] Guttman M, Amit I, Garber M, French C, Lin MF, Feldser D (2009). Chromatin signature reveals over a thousand highly conserved large non-coding RNAs in mammals. Nature.

[11] Pang KC, Frith MC, Mattick JS (2006). Rapid evolution of noncoding RNAs: lack of conservation does not mean lack of function. Trends Genet.

[12] Washietl S, Kellis M, Garber M (2014). Evolutionary dynamics and tissue specificity of human long noncoding RNAs in six mammals. Genome Res.

[13] Necsulea A, Soumillon M, Warnefors M, Liechti A, Daish T (2014). The evolution of lncrna repertoires and expression patterns in tetrapods. Nature.

[14] Managadze D, Lobkovsky AE, Wolf YI, Shabalina SA, Rogozin IB, Koonin EV (2013). The vast, conserved mammalian lincRNome. PLoS Comput Biol.

[15] Nitsche A, Rose D, Fasold M, Reiche K, Stadler PF. Comparison of splice sites reveals that long non-coding RNAs are evolutionarily well conserved. RNA. 2015 May; 21(5):801–12. doi:10.1261/rna.046342.114. Epub 2015 Mar 23. 10.1261/rna.046342.114 PMC4408788 25802408

[16] Hiller M, Findeiß S, Lein S, Marz M, Nickel C, Rose D (2009). Conserved introns reveal novel transcripts in *Drosophila melanogaster*. Genome Res.

[17] Chodroff RA, Goodstadt L, Sirey TM, Oliver PL, Davies KE, Green ED, et al (2010). Long noncoding RNA genes: conservation of sequence and brain expression among diverse amniotes. Genome Biol.

[18] Wang KC, Chang HY (2011). Molecular mechanisms of long noncoding RNAs. Mol Cell.

[19] Tilgner H, Knowles DG, Johnson R, Davis CA, Chakrabortty S, Djebali S (2012). Deep sequencing of subcellular RNA fractions shows splicing to be predominantly co-transcriptional in the human genome but inefficient for lncRNAs. Genome Res.

[20] Louhichi A, Fourati A, Rebaï A (2011). IGD: a resource for intronless genes in the human genome. Gene.

[21] Lei H, Dias AP, Reed R (2011). Export and stability of naturally intronless mRNAs require specific coding region sequences and the TREX mRNA export complex. Proc Natl Acad Sci USA.

[22] Shabalina SA, Ogurtsov AY, Spiridonov AN, Novichkov PS, Spiridonov NA, Koonin EV (2010). Distinct patterns of expression and evolution of intronless and intron-containing mammalian genes. Mol Biol Evol.

[23] Marzluff WF, Wagner EJ, Duronio RJ (2008). Metabolism and regulation of canonical histone mRNAs: life without a poly(A) tail. Nat Rev Genet.

[24] Nakaya HI, Amaral PP, Louro R, Lopes A, Fachel AA, Moreira YB (2007). Genome mapping and expression analyses of human intronic noncoding RNAs reveal tissue-specific patterns and enrichment in genes related to regulation of transcription. Genome Biol.

[25] Louro R, Nakaya HI, Amaral PP, Festa F, Sogayar MC, da Silva AM (2007). Androgen responsive intronic non-coding RNAs. BMC Biol.

[26] Louro R, El-Jundi T, Nakaya HI, Reis EM, Verjovski-Almeida S (2008). Conserved tissue expression signatures of intronic noncoding RNAs transcribed from human and mouse loci. Genomics.

[27] Rinn JL, Euskirchen G, Bertone P, Martone R, Luscombe NM, Hartman S (2003). The transcriptional activity of human chromosome 22. Genes Dev.

[28] Reis EM, Nakaya HI, Louro R, Canavez FC, Flatschart AV (2004). Antisense intronic non-coding RNA levels correlate to the degree of tumor differentiation in prostate cancer. Oncogene.

[29] Beckedorff FC, Ayupe AC, Crocci-Souza R, Amaral MS, Nakaya HI, Soltys DT (2013). The intronic long noncoding RNA *ANRASSF1* recruits PRC2 to the *RASSF1A* promoter, reducing the expression of *RASSF1A* and increasing cell proliferation. PLoS Genet.

[30] Gardner EJ, Nizami ZF, Talbot Jr CC, Gall JG (2012). Stable intronic sequence RNA (sisRNA), a new class of noncoding RNA from the oocyte nucleus of *Xenopus tropicalis*. Genes Dev.

[31] Louro R, Smirnova AS, Verjovski-Almeida S (2009). Long intronic noncoding RNA transcription: expression noise or expression choice?. Genomics.

[32] Tahira AC, Kubrusly MS, Faria MF, Dazzani B, Fonseca RS, Maracaja-Coutinho V (2011). Long noncoding intronic RNAs are differentially expressed in primary and metastatic pancreatic cancer. Mol Cancer.

[33] Whitehead J, Pandey GK, Kanduri C (2009). Regulation of the mammalian epigenome by long noncoding RNAs. Biochim Biophys Acta.

[34] Paul IJ, Duerksen JD (1975). Chromatin-associated RNA content of heterochromatin and euchromatin. Mol Cell Biochem.

[35] Rodríguez-Campos A, Azorín F (2007). RNA is an integral component of chromatin that contributes to its structural organization. PLoS ONE.

[36] Mondal T, Rasmussen M, Pandey GK, Isaksson A, Kanduri C (2010). Characterization of the RNA content of chromatin. Genome Res.

[37] Magistri M, Faghihi MA, St. Laurent 3rd G, Wahlestedt C. Regulation of chromatin structure by long noncoding RNAs: focus on natural antisense transcripts. Trends Genet. 2012; 28:389–96. 10.1016/j.tig.2012.03.013 PMC3768148 22541732

[38] Schubert T, Längst G (2013). Changes in higher order structures of chromatin by RNP complexes. RNA Biol.

[39] Kapranov P, Cheng J, Dike S, Nix D, Duttagupta R, Willingham AT (2007). RNA maps reveal new RNA classes and a possible function for pervasive transcription. Science.

[40] Wang X, Arai S, Song X, Reichart D, Du K, Pascual G (2008). Induced ncRNAs allosterically modify RNA-binding proteins *in cis* to inhibit transcription. Nature.

[41] Hamilton RS, Davis I (2011). Identifying and searching for conserved RNA localisation signals. Methods Mol Biol.

[42] Dassi E, Quattrone A (2012). Tuning the engine: an introduction to resources on post-transcriptional regulation of gene expression. RNA Biol.

[43] Jung H, Gkogkas CG, Sonenberg N, Holt CE (2014). Remote control of gene function by local translation. Cell.

[44] Mercer TR, Wilhelm D, Dinger ME, Soldà G, Korbie DJ, Glazov EA (2011). Expression of distinct RNAs from 3’ untranslated regions. Nucleic Acids Res.

[45] Flynn RA, Almada AE, Zamudio JR, Sharp PA (2011). Antisense RNA polymerase II divergent transcripts are P-TEFb dependent and substrates for the RNA exosome. Proc Natl Acad Sci.

[46] Niazi F, Valadkhan S (2012). Computational analysis of functional long noncoding RNAs reveals lack of peptide-coding capacity and parallels with 3’utrs. RNA.

[47] Ebert MS, Sharp PA (2010). Emerging roles for natural microRNA sponges. Curr Biol.

[48] Bak RO, Mikkelsen JG (2014). miRNA sponges: soaking up miRNAs for regulation of gene expression. Wiley Interdiscip Rev RNA.

[49] Hentze MW, Preiss T (2013). Circular RNAs: splicing’s enigma variations. EMBO J.

[50] Sasaki YTF, Ideue T, Sano M, Mituyama T, Hirose T (2009). MEN *ε*/*β* noncoding RNAs are essential for structural integrity of nuclear paraspeckles. Proc Natl Acad Sci USA.

[51] Sunwoo H, Dinger ME, Wilusz JE, Amaral PP, Mattick JS, Spector DL (2009). MEN *ε*/*β* nuclear-retained non-coding RNAs are up-regulated upon muscle differentiation and are essential components of paraspeckles. Genome Res.

[52] Mao YS, Sunwoo H, Zhang B, Spector DL (2011). Direct visualization of the co-transcriptional assembly of a nuclear body by noncoding RNAs. Nat Cell Biol.

[53] Hutchinson J, Ensminger AW, Clemson CM, Lynch CR, Lawrence JB, Chess A (2007). A screen for nuclear transcripts identifies two linked noncoding RNAs associated with SC35 splicing domains. BMC Genomics.

[54] Stadler PF, Ferreira CE, Miyano S, Stadler PF, editors (2010). Evolution of the long non-coding RNAs MALAT1 and MEN *β*/ *ε*. Advances in Bioinformatics and Computational Biology, 5th Brazilian Symposium on Bioinformatics. Lecture Notes in Computer Science, vol.6268.

[55] Chung S, Nakagawa H, Uemura M, Piao L, Ashikawa K, Hosono N (2011). Association of a novel long non-coding RNA in 8q24 with prostate cancer susceptibility. Cancer Sci.

[56] Hackermüller J, Reiche K, Otto C, Hösler N, Blumert C, Brocke-Heidrich K (2014). Cell cycle, oncogenic and tumor suppressor pathways regulate numerous long and macro non-protein coding RNAs. Genome Biol.

[57] Seidl CIM, Stricker SH, Barlow DP (2006). The imprinted Air ncRNA is an atypical RNAPII transcript that evades splicing and escapes nuclear export. EMBO J.

[58] Stricker SH, Steenpass L, Pauler FM, Santoro F, Latos PA, Huang R (2008). Silencing and transcriptional properties of the imprinted Airn ncRNA are independent of the endogenous promoter. EMBO J.

[59] Redrup L, Branco MR, Perdeaux ER, Krueger C, Lewis A, Santos F (2009). The long noncoding RNA Kcnq1ot1 organises a lineage-specific nuclear domain for epigenetic gene silencing. Development.

[60] Nordström KJ, Mirza MA, Almén MS, Gloriam DE, Fredriksson R, Schiöth HB (2009). Critical evaluation of the FANTOM3 non-coding RNA transcripts. Genomics.

[61] Engelhardt J, Stadler PF (2012). Hidden treasures in unspliced est data. Theory Biosci.

[62] Greenbaum D, Colangelo C, Williams K, Gerstein M (2003). Comparing protein abundance and mRNA expression levels on a genomic scale. Genome Biol.

[63] Gry M, Rimini R, Strömberg S, Asplund A, Pontén F, Uhlén M (2009). Correlations between RNA and protein expression profiles in 23 human cell lines. BMC Genomics.

[64] Thorrez L, Tranchevent LC, Chan HJ, Moreau Y, Schuit F (2010). Detection of novel 3’ untranslated region extensions with 3’ expression microarrays. BMC Genomics.

[65] Proudfoot NJ (2011). Ending the message: poly(A) signals then and now. Genes Dev.

[66] Li Y, Sun Y, Fu Y, Li M, Huang G, Zhang C (2012). Dynamic landscape of tandem 3’ UTRs during zebrafish development. Genome Res.

[67] Lianoglou S, Garg V, Yang JL, Leslie CS, Mayr C (2013). Ubiquitously transcribed genes use alternative polyadenylation to achieve tissue-specific expression. Genes Dev.

[68] Shiraki T, Kondo S, Katayama S, Waki K, Kasukawa T, Kawaji H (2003). Cap analysis gene expression for high-throughput analysis of transcriptional starting point and identification of promoter usage. Proc Natl Acad Sci U S A.

[69] The FANTOM Consortium and others (2014). A promoter-level mammalian expression atlas. Nature.

[70] Ørom UA, Shiekhattar R (2013). Long noncoding RNAs usher in a new era in the biology of enhancers. Cell.

[71] Lai F, Shiekhattar R (2014). Enhancer RNAs: the new molecules of transcription. Curr Opin Genet Dev.

[72] Lam MT, Li W, Rosenfeld MG, Glass CK (2014). Enhancer RNAs and regulated transcriptional programs. Trends Biochem Sci.

[73] Kim TK, Hemberg M, Gray JM (2015). Enhancer RNAs: a class of long noncoding RNAs synthesized at enhancers. Cold Spring Harb Perspect Biol.

[74] Andersson R, Gebhard C, Miguel-Escalada I, Hoof I, Bornholdt J, Boyd M (2014). An atlas of active enhancers across human cell types and tissues. Nature.

[75] Ohmiya H, Vitezic M, Frith MC, Itoh M, Carninci P, Forrest AR (2014). RECLU: a pipeline to discover reproducible transcriptional start sites and their alternative regulation using capped analysis of gene expression (cage). BMC Genomics.

[76] Derrien T, Johnson R, Bussotti G, Tanzer A, Djebali S, Tilgner H (2012). The GENCODE v7 catalog of human long noncoding RNAs: analysis of their gene structure, evolution, and expression. Genome Res.

[77] Hoffman MM, Ernst J, Wilder SP, Kundaje A, Harris RS, Libbrecht M, et al (2013). Integrative annotation of chromatin elements from encode data. Nucleic Acids Res.

[78] Devor EJ, Moffat-Wilson KA (2003). Molecular and temporal characteristics of human retropseudogenes. Hum Biol.

[79] Yu Z, Morais MDIvanga, Harrison PM (2007). Analysis of the role of retrotransposition in gene evolution in vertebrates. BMC Bioinformatics.

[80] Washietl S, Hofacker IL, Stadler PF (2005). Fast and reliable prediction of noncoding RNAs. Proc Natl Acad Sci USA.

[81] Gruber AR, Findeiß S, Washietl S, Hofacker IL, Stadler PF (2010). RNAz 2.0: improved noncoding RNA detection. Pac Symp Biocomput.

[82] Washietl S, Findeiß S, Müller S, Kalkhof S, von Bergen M, Hofacker IL, et al (2011). RNAcode: robust prediction of protein coding regions in comparative genomics data. RNA.

[83] ENCODE Project Consortium (2012). An integrated encyclopedia of dna elements in the human genome. Nature.

[84] Siepel A, Bejerano G, Pedersen JS, Hinrichs A, Hou M, Rosenbloom K (2005). Evolutionarily conserved elements in vertebrate, insect, worm, and yeast genomes. Genome Res.

[85] Hubisz MJ, Pollard KS, Siepel A (2011). Phast and rphast: phylogenetic analysis with space/time models. Brief Bioinform.

[86] Mercer TR, Dinger ME, Bracken CP, Kolle G, Szubert JM, Korbie DJ (2010). Regulated post-transcriptional RNA cleavage diversifies the eukaryotic transcriptome. Genome Res.

[87] Eberle AB, Hessle V, Helbig R, Dantoft W, Gimber N, Visa N (2010). Splice-site mutations cause rrp6-mediated nuclear retention of the unspliced RNAs and transcriptional down-regulation of the splicing-defective genes. PLoS ONE.

[88] Karolchik D, Hinrichs AS, Furey TS, Roskin KM, Sugnet CW, Haussler D (2004). The ucsc table browser data retrieval tool. Nucleic Acids Res.

[89] Hawkins JD (1988). A survey on intron and exon lengths. Nucleic Acids Res.

[90] Hong X, Scofield DG, Lynch M (2006). Intron size, abundance, and distribution within untranslated regions of genes. Mol Biol Evol.

[91] Horton R, Gibson R, Coggill P, Miretti M, Allcock RJ, Almeida J (2008). Variation analysis and gene annotation of eight mhc haplotypes: the mhc haplotype project. Immunogenetics.

[92] Hazkani-Covo E, Zeller R, Martin W (2010). Molecular poltergeists: Mitochondrial DNA copies (numts) in sequenced nuclear genomes. PLoS Genet.

[93] Asin-Cayuela J, Gustafsson CM (2007). Mitochondrial transcription and its regulation in mammalian cells. Trends Biochem Sci.

[94] Tsuji J, Frith MC, Tomii K, Horton P (2012). Mammalian NUMT insertion is non-random. Nucleic Acids Res.

[95] Pruitt KD, Tatusova T, Maglott DR (2007). NCBI reference sequences (RefSeq): a curated non-redundant sequence database of genomes, transcripts and proteins. Nucleic Acids Res.

[96] Harrow J, Frankish A, Gonzalez JM, Tapanari E, Diekhans M, Kokocinski F (2012). Gencode: the reference human genome annotation for the encode project. Genome Res.

[97] Kawaji H, Severin J, Lizio M, Waterhouse A, Katayama S, Irvine KM (2009). The fantom web resource: from mammalian transcriptional landscape to its dynamic regulation. Genome Biol.

[98] Quinlan AR, Hall IM (2010). Bedtools: a flexible suite of utilities for comparing genomic features. Bioinformatics.

[99] R Core Team (2013). R: A Language and Environment for Statistical Computing.

[100] Goecks J, Nekrutenko A, Taylor J, Galaxy Team (2010). Galaxy: a comprehensive approach for supporting accessible, reproducible, and transparent computational research in the life sciences. Genome Biol.

[101] Kent WJ, Sugnet CW, Furey TS, Roskin KM, Pringle TH, Zahler AM (2002). The human genome browser at ucsc. Genome Res.

[102] Kawaji H, Severin J, Lizio M, Forrest AR, van Nimwegen E, Rehli M (2011). Update of the fantom web resource: from mammalian transcriptional landscape to its dynamic regulation. Nucleic Acids Res.

[103] Chiaromonte F, Yap V, Miller W. Scoring pairwise genomic sequence alignments, 2002. In: Russ B. Altman, Keith A. Dunker, Lawrence Hunter, Kevin Lauderdale, Teri E. Klein, editors. Pac Symp Biocomput. vol. 7. USA: World Scientific Publishing Co Pte Ltd: 2002. p. 115–26. 10.1142/9789812799623_0012 11928468

[104] Schwartz S, Kent WJ, Smit A, Zhang Z, Baertsch R, Hardison RC (2003). Human-mouse alignments with blastz. Genome Res.

