# Supplementary material for: Evolution of the unspliced transcriptome
Source: BMC Evol Biol. 2015 Aug 20;15:166. doi: 10.1186/s12862-015-0437-7 (PMC4546029; doi:10.1186/s12862-015-0437-7)
Supplement: Additional file 1 — Supplement. Supplemental information about the methods and results. Includes example regions showing unspliced ESTs cluster detected to be especially interesting for various reasons. Additional data – UCSC Track Hubs; A track hub for the UCSC genome browser was created. It consists of a track of all unspliced EST cluster as well as tracks for the different classes of unspliced EST cluster for hg19 and mm10. Additional bed files for intermediate results of the analysis are available on request. The track can be accessed at: http://www.bioinf.uni-leipzig.de/~jane/data/uESTHub/hub.txt. Additional information how to use it can be found in the UCSC manual pages: http://www.genome.ucsc.edu/goldenPath/help/hgTrackHubHelp.html. (PDF 1587 kb) [file 12862_2015_437_MOESM1_ESM.pdf]

Evolution of the Unspliced Transcriptome - Supplement

Jan Engelhardt and Peter F. Stadler

July 7, 2015

Additional data

ESTs "within range" of RefSeq genes

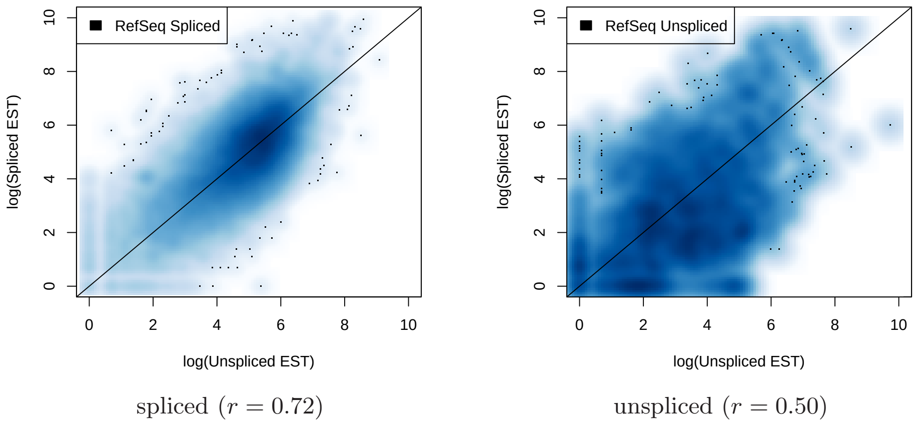

Figure S1: Scatter plots of the relative abundance of human spliced and unspliced ESTs within the range of both spliced and unspliced RefSeq genes show only a relatively poor correlation.

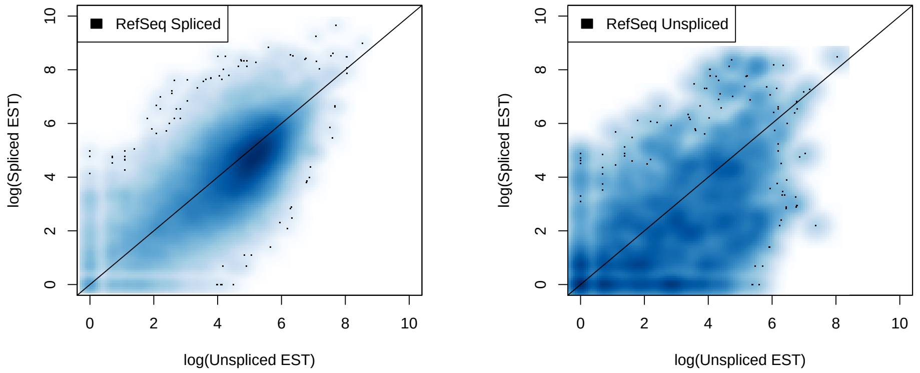

spliced ($r = 0.72$) unspliced ($r = 0.48$)

Figure S2: Scatter plots of the relative abundance of mouse spliced and unspliced ESTs within the range of both spliced and unspliced RefSeq genes show only a relatively poor correlation.

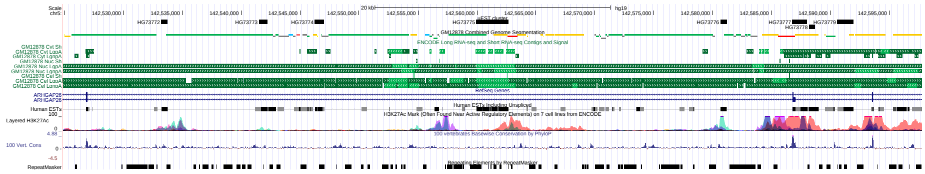

Figure S3: ARHGAP26 (Rho GTPase activating protein) is a long protein-coding gene on chromosome 5 in human, stretching over appr. 46kb (blue line with arrows). Between the 20th and 21st exon we find a unspliced EST (uEST) cluster (HG73775) with a length of 2,700 nt (black box). Additional evidence for functional importance is given by ENCODE data. Long RNAseq transcripts antisense to the protein-coding gene have been reported (green bar, white arrows depict the reading direction). Additionally the region upstream of the uEST cluster is classified as transcription start [1] (the tiny red bar enclosed by yellow and green bars below the EST track), assuming the reading direction predicted by RNAseq. There is also an enrichment of the histone modification H3K27Ac around the uEST cluster (the colored transparent overlays near the bottom). This modification is often found near active regulatory elements.

Table S1: Summary of human EST data. Analysis of the mRNA annotation track of hg19 and mm10 downloaded from the UCSC genome browser in November 2013. The numbers indicate successive filters. Which means just the mRNAs which do not have more than one block are included in the number of mRNAs with more than 60nt in length.

Type	Human	Mouse
all mRNAs	399,812	329,819
> 1 block	247,389	188,945
$< 60nt$ lenth	93,301	91,182
unspliced mRNAs	59,122	49,665

Unspliced mRNAs

The track “all_mrna” was downloaded for Human and Mouse from UCSC genome browser on 15th of November 2013 and 18th of November, respectively. We removed all mRNAs with more than one block, since we were interested in unspliced ones. A large part of the remaining data consisted of rather short mRNAs. One of the shortest mini-protein known is an artificially designed 20-residue construct [2]. Furthermore one can assume that an actual protein needs some additional UTR sequences on the genomic level. Considering these facts it is very unlikely to find many true positive mRNAs with a length shorter than 60 nucleotides. Consequently we remove all mRNAs shorter than this length from our data set, see Tab. S1.

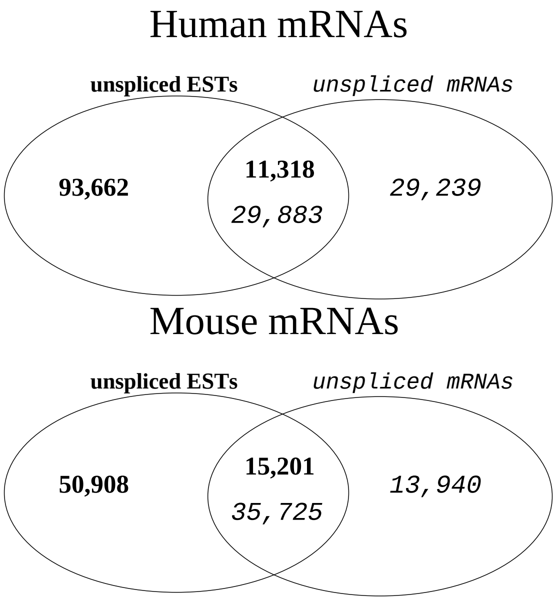

Figure S4: Overlap of unspliced EST (uEST) cluster with annotated unspliced mRNAs in human and mouse. Bold numbers indicate the amount of uEST cluster while non-bold numbers indicate the amount of unspliced mRNAs. Numbers depicted in the overlap of both entities refer to uEST overlapping unspliced mRNAs and vice versa.

50,498 of 59,122 (35,430 of 49,665 in mouse) annotated ‘unspliced mRNAs’ are located within the extended RefSeq regions, of which 41,361 (16,303 in mouse) overlap RefSeq exons (including annotated unspliced RefSeq genes). We find that 51% (72% in mouse) of the unspliced mRNAs overlap with about 11% (23% in mouse) of the unspliced ESTs, see Fig. S4. Among these unspliced mRNAs there are several famous transcripts, cf. Tab. S2.

MALAT-1, for instance, appears among the loci with the highest coverage of unspliced ESTs. Together with NEAT1, which is located in the same genomic region, it belongs to a class of long nuclear retained transcripts involved in the organization of nuclear speckles [4, 5], see also [6] and the references therein. Other examples, such as KCNQ1OT1 [7] and PTCSC3 [8] are clearly visible in our data, albeit with moderate coverage. Unspliced ESTs are also reported as parts of known spliced non-coding transcripts, in particular those with very long exons such as XIST [9]. In other cases, such as TUG1 [10], we observe predominantly unspliced ESTs that cover (nearly) the entire primary transcript, even though the genomic location is annotated by the spliced forms.

Table S2: Coverage of well-known long ncRNAs by unclustered unspliced ESTs (# uESTs). The first group is annotated as predominantly unspliced. The second group has annotated spliced isoforms. Coordinates taken by `lncrna db`[3].

Gene	Chr.		approx.loc.		# uESTs	
Species	Human	Mouse	Human	Mouse	Human	Mouse
MALAT1	11	19	65.27mb	5.80mb	16,917	864
NEAT1	11	19	65.19mb	5.82mb	2,848	876
KCNQ1OT1	11	7	2.66mb	143.21mb	96	192
PTCSC3	14	-	36.60mb	-	10	-
XIST	X	X	73.04mb	103.46mb	1,341	314
TUG1	22	11	31.37mb	3.64mb	907	227
HOTAIR	12	15	54.36mb	102.94mb	22	3
AIR	-	17	-	12.74mb	-	218
ANRIL	9	-	21.99mb	-	120	-

Independent UTR-derived RNAs

Humand and Mouse CAGE data

Cell-type specific human data

The TSS peaks in 156 primary human cell lines predicted by *Ohmiya et al.* [11] were downloaded from the FANTOM5 webserver. The unspliced EST cluster and RefSeq genes were the same as in the whole analysis. For the long noncoding RNAs a dataset based on the GENCODE v14 annotation but filtered for a higher reliability by *Nitsche et al.* [12].

The analysis was the same for all three data sets. We filtered for elements that overlaped with a predicted TSS on the forward strand in their first 84 nucleotides or a TSS from the reverse strand on their last 84 nt. This number was chosen to be able to compare the results to our first analysis in 2012 [13].

The number of cell lines for which an element was predicted to be expressed is visualized in Supp. Fig. S5.

Table S3: Distribution of unspliced EST clusters with a predicted TSS peak in their putative 5' region through the different classes. "Forward" are cluster on the genomic forward strand, "Reverse" are on the backward strand.

Species	Human			Mouse		
Type	Forward	Reverse	All	Forward	Reverse	All
UT	281	280	2,459	190	224	1,601
5'R	401	415	1,583	440	441	1,502
5'PIN	1,060	1,021	4,315	785	731	2,878
TIN	1,464	1,375	38,803	722	762	20,020
3'PIN	298	270	5,710	176	177	3,040
rI	152	145	715	94	87	477
3'R	300	275	2,821	238	233	3,116
DT	143	138	3,950	92	98	2,600

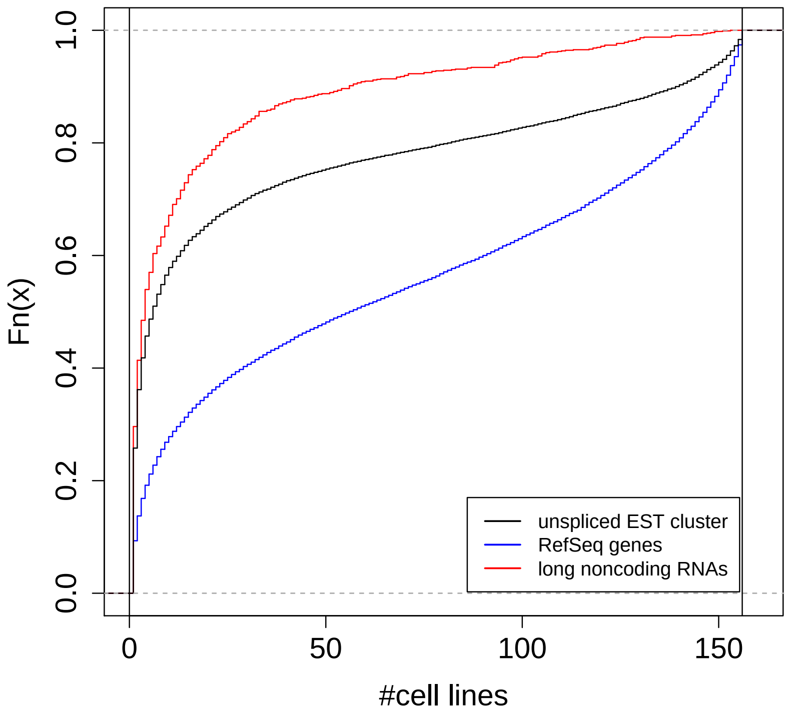

Figure S5: The plot shows the cummulative distribution of genes or transcripts that have a predicted TSS in at least a given number of different cell lines. Long noncoding RNAs are taken from GENCODE.

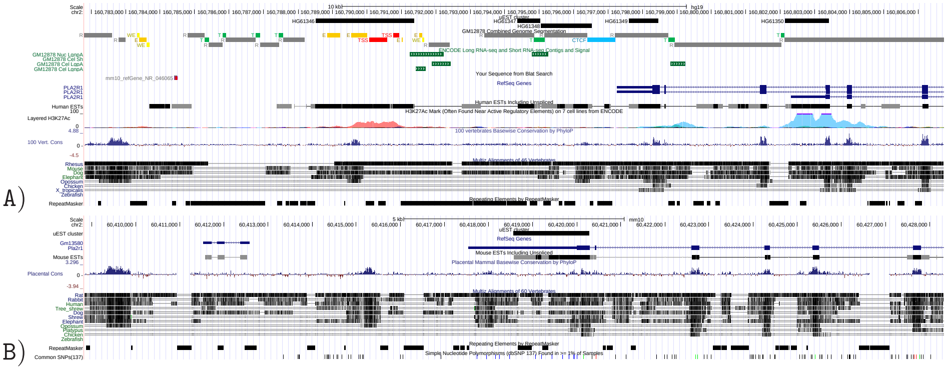

Figure S6: A)PLA2R1 and downstream region on chromosome 2 in human is an example of a candidate for a new lncRNA. The importance of the unspliced EST (uEST) cluster HG61346 is supported by various evidence. Transcripts antinsense to the neighboring gene PLA2R1 have been detected by long RNAseq (green bars, white arrows depict the reading direction). The uEST overlaps with a region annotated as transcription start site by analysing the chromatin structure (red bar named TSS). In the middle of the uEST cluster there is a siginificant peak of evolutionary conservation visible (blue histogram). B) PLA2R1 and downstream region with lncRNA Gm13880 on chromosome 2 is the homologous region in mouse. In contrast to human there is an antisense and spliced long non-coding RNA annotated downstream of PLA2R1. A small part of its sequence (76nt) can be found in the human genome using *BLAT*. To know the exact structure of the lncRNA additional investigation, most likely by experimental methods is necessary.

Conserved secondary structure on forward strand

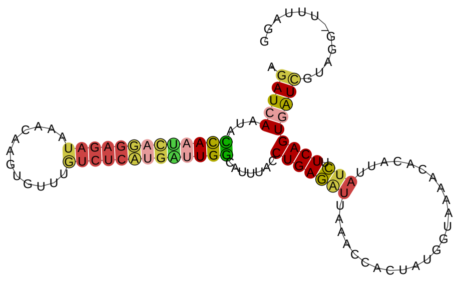

Conserved secondary structure on reverse strand

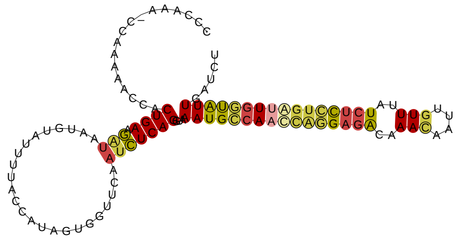

Figure S7: Conserved secondary structure elements providing one of the best `RNAz` scores. The multiple genome alignment of the region hg19.chr9:66,766,331 66,766,440 contained sequences from diverse mammals, including Pteropus vampyrus and Loxodonta africana. The upper picture is the structure on the forward strand while the lower one is on the reverse strand. Both have a p-value better than 0.99. The color code, from red to ochre and green indicates that 1, 2, or 3 different types of basepairs are observed in the corresponding alignment columns, unsaturated colors indicate basepairs that cannot be formed by 1 or 2 of the 6 sequences in the alignment. Substitutions in stem regions are indicated by circles.

Overlap with ENCODE RNA-seq data

We investigated the overlap of the unspliced EST cluster with the published ENCODE RNA-seq data. 96.6% of all human unspliced EST cluster are supported by RNA-seq data, defined as overlapping with at least 10 reads of at least 1 RNA-seq library. In most classes only a small fraction of the cluster is not supported by RNA-seq data. Only the class IGR stands out with 15.4% of unspliced EST cluster without RNA-seq evidence, all numbers can be found in Supp. Tab. S4.

Table S4: The table shows the amount of unspliced EST cluster per class that are not supported by ENCODE RNA-seq data. An cluster is supported if it has an overlap with at least 10 reads in at least one RNA-seq library. The numbers in the table represent the cluster that do not fulfill this requirement.

Type	UT	5R	TEX	PIN5	TIN	PIN3
hg19	137 (5.6%)	34 (2.1%)	319 (2.6%)	40 (1%)	791 (2%)	65 (1.1%)
Type	rI	3R	DT	IGR		
hg19	4 (0.6%)	52 (1.8%)	139 (3.5%)	1723 (15.4%)		

Conservation

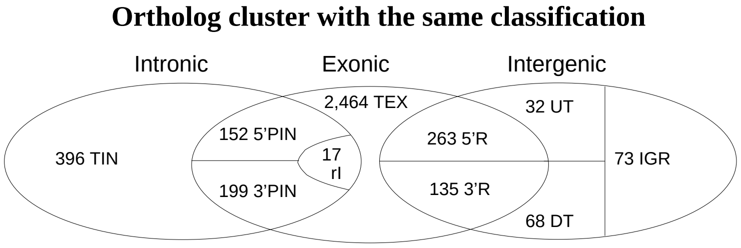

Figure S8: Distribution of the classes in pairs between homologous cluster which are classified in the same class in human and mouse. 1,495 cluster are assigned to "NO_CLASS".

Conserved totally intronic transcript on chr.7 in Human

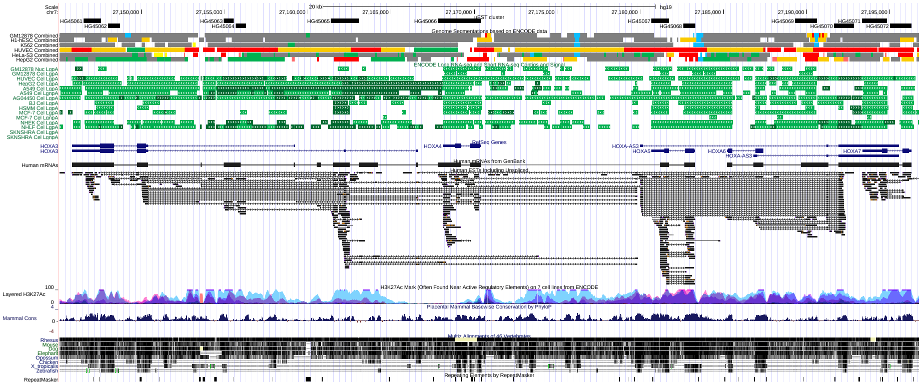

Conserved totally intronic transcript on chr.6 in Mouse

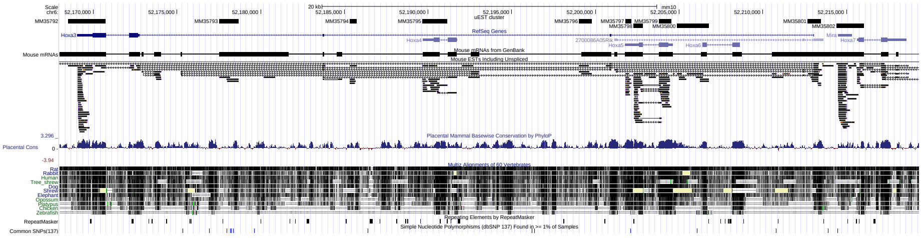

Figure S9: Example of conserved totally intronic unspliced EST (uEST) cluster. The TINs HG45063/HG45064 and MM35793 have not been reported previously. The overlapping gene is HOXA3. The genomic loci is rather complex as one can see by the large number of differently connected ESTs and mRNAs. The uEST cluster HG45065 has been recently described as beeing an apoptosis repressor in certan cell lines and was called HOXA-AS2 [14]. The independence of HG45063/HG45064 can not be determined without additional experiments. It might be a splice variant of HOXA-AS2 or even of HOXA-AS3. In fact they could turn out to be a single large antisense ncRNA, maybe depending on the used gene definition. Nevertheless, transcription is supported by chromatin marks (cell lines HeLa-S3 and HepG2) and antisense RNAseq data (see caption of Fig. S6).

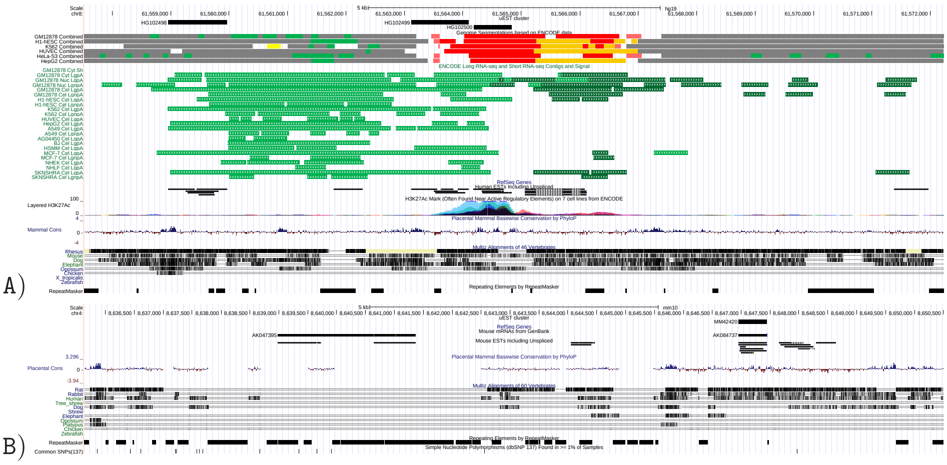

Figure S10: `A`) Conserved intergenic unspliced EST cluster on chromosome 8 in human. The cluster HG102499 and HG102500 in human are homolog to MM42420 in mouse. The distrubtion of uESTs in the flanking region indicates a larger spliced transcript with at least three exons. In human there is additional evidence by chromatin marks and RNAseq data (see caption of Fig. S6). `B`) Conserved intergenic unspliced EST cluster on chromosome 4 in mouse is homologous to the ones in `A`

Table S5: Mean conservation `phastConst` conservation scores stratified for the different types of unspliced EST clusters. Scores refer to the phastCons100way track for human and phastCons60way for mouse. (Only IGR cluster without a RNAcode signal have been included)

Type	UT	5R	TEX	PIN5	TIN	PIN3
hg19	0.113	0.343	0.332	0.249	0.093	0.278
mm10	0.193	0.395	0.371	0.287	0.161	0.335
Type	rI	3R	DT	IGR	∅	
hg19	0.285	0.260	0.107	0.142	0.237	
mm10	0.356	0.322	0.189	0.272	0.297	
Type	Exon	Intron	Gene	Genome		
hg19	0.602	0.0881	0.109	0.102		
mm10	0.665	0.129	0.156	0.150		

Table S6: Mean conservation phastConst conservation scores stratified for the different types TEX unspliced EST clusters. Scores refer to the phastCons100way track for human and phastCons60way for mouse.

Type	TEX overlapping CDS	TEX completly in CDS	TEX not overlapping CDS
hg19	0.382 (9,385/12,395)	0.571 (1,446/12,395)	0.203 (3,010/12,395)
mm10	0.407 (9,545/12,751)	0.569 (820/12,751)	0.259 (3,206/12,751)

References

[1] Hoffman, M.M., Ernst, J., Wilder, S.P., Kundaje, A., Harris, R.S., Libbrecht, M., Giardine, B., Ellenbogen, P.M., Bilmes, J.A., Birney, E., Hardison, R.C., Dunham, I., Kellis, M., Noble, W.S.: Integrative annotation of chromatin elements from encode data. Nucleic Acids Research **41**(2), 827–841 (2013)

[2] Neidigh, J.W., Fesinmeyer, R.M., H, A.N.: Designing a 20-residue protein. Nature Structural Biology **9**, 425–430 (2002)

[3] Amaral, P.P., Clark, M.B., Gascoigne, D.K., Dinger, M.E., Mattick, J.S.: lncrnadb: a reference database for long noncoding rnas. Nucleic Acids Res **39**(Database issue), 146–151 (2011)

[4] Hutchinson, J., Ensminger, A.W., Clemson, C.M., Lynch, C.R., Lawrence, J.B., Chess, A.: A screen for nuclear transcripts identifies two linked noncoding RNAs associated with SC35 splicing domains. BMC Genomics **8**, 39 (2007)

[5] Lin, R., Roychowdhury-Saha, M., Black, C., Watt, A.T., Marcusson, E.G., Freier, S.M., Edgington, T.S.: Control of RNA processing by a large noncoding RNA over-expressed in carcinomas. FEBS Lett. **585**, 671–671 (2011)

[6] Stadler, P.F.: Evolution of the long non-coding RNAs MALAT1 and MENβ/ϵ. In: Ferreira, C.E., Miyano, S., Stadler, P.F. (eds.) Advances in Bioinformatics and Computational Biology, 5th Brazilian Symposium on Bioinformatics. Lecture Notes in Computer Science, vol. 6268, pp. 1–12. Springer, Heidelberg (2010)

[7] Redrup, L., Branco, M.R., Perdeaux, E.R., Krueger, C., Lewis, A., Santos, F., Nagano, T., Cobb, B.S., Fraser, P., Reik, W.: The long noncoding RNA Kcnq1ot1 organises a lineage-specific nuclear domain for epigenetic gene silencing. Development **136**, 525–530 (2009)

[8] He, H., Nagy, R., Liyanarachchi, S., Jiao, H., Li, W., Suster, S., Kere, J., de la Chapelle, A.: A susceptibility locus for papillary thyroid carcinoma on chromosome 8q24. Cancer Res. **69**, 625–631 (2009)

[9] Elisaphenko, E.A., Kolesnikov, N.N., Shevchenko, A.I., Rogozin, I.B., Nesterova, T.B., Brockdorff, N., Zakian, S.M.: A dual origin of the *Xist* gene from a protein-coding gene and a set of transposable elements. PLoS One **3**, 2521 (2008)

[10] Young, T.L., Matsuda, T., Cepko, C.L.: The noncoding RNA taurine upregulated gene 1 is required for differentiation of the murine retina. Current Biology **15**, 501–512 (2005)

[11] Ohmiya, H., Vitezic, M., Frith, M.C., Itoh, M., Carninci, P., Forrest, A.R., Hayashizaki, Y., Lassmann, T., *et al.*: RECLU: a pipeline to discover reproducible transcriptional start sites and their alternative regulation using capped analysis of gene expression (cage). BMC genomics **15**(1), 269 (2014)

[12] Nitsche, A., Rose, D., Fasold, M., Reiche, K., Stadler, P.F.: Comparison of splice sites reveals that long non-coding RNAs are evolutionarily well conserved. RNA (2015). in press

[13] Engelhardt, J., Stadler, P.F.: Hidden treasures in unspliced est data. Theory Biosci **131**(1), 49–57 (2012)

[14] Zhao, H., Zhang, X., Frazao, J.B., Condino-Neto, A., Newburger, P.E.: Hox antisense lincrna hoxa-as2 is an apoptosis repressor in all trans retinoic acid treated nb4 promyelocytic leukemia cells. Journal of Cellular Biochemistry **114**(10), 2375–2383 (2013)
